# Patterns of synaptic loss in human amyotrophic lateral sclerosis spinal cord: a clinicopathological study

**DOI:** 10.1186/s40478-023-01616-8

**Published:** 2023-07-25

**Authors:** Oumayma Aousji, Simone Feldengut, Stefano Antonucci, Michael Schön, Tobias M. Boeckers, Jakob Matschke, Christian Mawrin, Albert C. Ludolph, Kelly Del Tredici, Francesco Roselli, Heiko Braak

**Affiliations:** 1 Department of Neurology, Center for Biomedical Research (ZBF), Ulm University, Helmholtzstraße 8/1, 89081 Ulm, Germany; 2 Clinical Neuroanatomy, Department of Neurology, Center for Biomedical Research (ZBF), Ulm University, Helmholtzstraße 8/1, 89081 Ulm, Germany; 3 Institute for Anatomy and Cell Biology, Ulm University, Ulm, Germany; 4 German Center for Neurodegenerative Diseases (DZNE), Ulm, Germany; 5 Institute of Neuropathology, University Medical Center Hamburg-Eppendorf, Hamburg, Germany; 6 Institute of Neuropathology, Otto-Von-Guericke-University, Magdeburg, Germany

**Keywords:** Amyotrophic lateral sclerosis, Phosphorylated TDP-43 (pTDP-43), Human spinal cord, Synaptic loss, Synaptophysin

## Abstract

Amyotrophic Lateral Sclerosis (ALS) is mainly characterized by the degeneration of corticospinal neurons and spinal α-motoneurons; vulnerable cells display prominent pTDP-43 inclusions. Evidence gathered from genetics, murine models, and iPSC-derived neurons point to the early involvement of synapses in the disease course and their crucial role in the pathogenic cascade. However, pathology studies, with specimens from large post-mortem cohorts, mapping the pattern of synaptic disturbances over clinical and neuropathological hallmarks of disease progression, are currently not available. Thus, the appearance and progression of synaptic degeneration in human ALS patients are currently not known, preventing a full validation of the murine and in vitro models. Here, we investigated the loss of synaptophysin-positive terminals in cervical, thoracic, and lumbar spinal cord samples from a retrospective cohort of n = 33 ALS patients and n = 8 healthy controls, and we correlated the loss of synapses against clinicodemographic features and neuropathological ALS stage. We found that, although dorsal and intermediate spinal cord laminae do not lose synapses, ALS patients displayed a substantial but variable loss of synapses in the ventral horn of lumbar and cervical spinal cord. The amount of synaptic loss was predicted by disease duration, by the clinical site of onset, and by the loss of α-motoneurons, although not by the fraction of pTDP-43-immunopositive α-motoneurons. Taken together, our findings validate the synaptic pathology observed in other models and suggest that pathogenic pathways unfolding in the spinal microenvironment are critical to the progressive disassembly of local synaptic connectivity.

**Supplementary Information:**

The online version contains supplementary material available at 10.1186/s40478-023-01616-8.

## Introduction

Amyotrophic lateral sclerosis (ALS) is defined by the primary involvement of corticospinal projection neurons in the motor cortex and α-motoneurons (MN) in the ventral horn of spinal cord and lower brainstem, with a progressive course and poor prognosis within 4–5 years from diagnosis [17]. ALS neuropathology is characterized by inclusions that are immunopositive for phosphorylated TAR DNA-binding protein (pTDP-43) in the cytoplasm of affected neurons in the cortex and spinal cord [3, 8, 27]. The appearance of pTDP-43 inclusions displays a pattern of an apparent timely and sequential propagation throughout cortical and subcortical structures during disease progression [6, 29].

Disruption of synaptic structures was shown to be a common and core feature of ALS. Pathology studies identified a decrease in the number of presynaptic terminals in post-mortem human spinal cord [9, 21, 33] as well as in the cerebral cortex [19]. Furthermore, the composition of synapses is altered in the ALS cortex and spinal cord [22]. Early changes in synaptic structural and functional integrity also have been identified in ALS animal models that affect the post-synaptic structures of excitatory and inhibitory synapses [2, 4], thereby supporting the concept that synaptic alterations may be concomitant or even precede the appearance of MN dysfunction and loss. Interestingly, reduced synaptic numbers and structural abnormalities of the remaining contacts also have been described in iPSC-derived motoneurons carrying diverse ALS-related mutations [11].

Nevertheless, possible drivers and modifiers of the synaptic loss in human ALS patients remain unclear, and several models have been proposed: synaptic loss may be driven by the burden of pTDP-43 pathology itself or, alternatively, may be related to the death of MN and other spinal cord neurons. Furthermore, it is unclear whether synaptic loss appears early in the disease course and contributes to MN vulnerability or accrues over time as MN degenerate. Third, the relationship between synaptic loss in the spinal cord and disease progression in the brain is unknown. Although pTDP-43 inclusions develop in different brain structures in a stereotypical pattern during disease progression and permit neuropathological staging [6, 8, 15], according to the proposed staging model, disruption of spinal cord synapses should be seeded from the motor cortex through the corticospinal tract (CST) early (stage I) and then progress independently of the involvement of other cortical areas that have limited or no direct projections to the spinal cord.

To date, these hypotheses have not been subject to experimental verification in large human specimen cohorts, and they remain difficult to test in animal models because of the different projections of the CST in rodents compared to humans, mainly directed toward sensory circuits in the former and more directed toward motor circuits in the latter [34]. Thus, the elucidation of the relationship between pTDP-43 and synaptic pathology in the spinal cord, on the one hand, and clinicopathological features, including the neuropathological brain stage [8], on the other, is a critical testbed for competing models of ALS pathophysiology. Here, we have subjected these hypotheses to investigation in a cohort of brain and spinal cord pathology samples from ALS patients, and we identified disease duration and the site of clinical onset as major predictors of synaptic loss in the ventral spinal cord.

## Materials and methods

### Study cohort

Autopsy tissue from human brains was collected at all three participating university hospitals, with informed consent of patients and/or their next of kin and approval of local institutional review boards. The retrospective study was conducted in compliance with the declaration of Helsinki and German federal as well as state law governing human tissue usage. A total of thirty-three (n = 33) post-mortem cases with a clinically and neuropathologically confirmed diagnosis of ALS [1, 24] from the Ulm University Tissue Bank and Department of Anatomy, as well as from the Institutes of Neuropathology at the University of Hamburg-Eppendorf and University of Magdeburg, were staged (HB, KDT) according to a previously published protocol [8]. The spinal cord samples available for each patient or control are reported in Table 1. None had known familial ALS according to clinical interviews and were therefore considered to be “sporadic” cases. Genotyping was not available for any of the individuals. Eight (n = 8) without neurodegenerative or neurological diseases were included as controls. Demographic, clinical, and neuropathological staging data of the cases examined are summarized in Table 1.

**Table 1:** Clinical data of the n = 8 controls and n = 33 ALS cases studied

ALS stage	Case number	Age at death (years)	Gender	Disease duration (years)	Clinical onset	Anatomical region of the spinal cord
*Controls*						
0	1	93	M	0	0	L
0	2	75	F	0	0	L
0	3	69	F	0	0	L
0	4	79	M	0	0	L, T
0	5	73	F	0	0	C
0	6	73	F	0	0	T
0	7	84	F	0	0	T
0	8	75	M	0	0	T
*ALS*						
1	9	48	M	0.9	UE	L, C, T
1	10	69	M	1.9	LE	L, C, T
1	11	51	M	NA	NA	C, T
2	12	60	M	2	UE	L, C, T
2	13	64	F	2.3	UE	L, C, T
2	14	62	F	3.3	LE	L, C, T
2	15	64	M	1.8	LE	L, C, T
2	16	53	M	3.3	LE	L, C, T
2	17	42	F	2.3	LE	L, C, T
2	18	56	M	1.9	LE	L, C, T
2	19	80	M	NA	NA	L
2	20	56	F	NA	NA	C, T
2	21	NA	NA	NA	NA	C, T
2	22	NA	NA	NA	NA	T
3	23	43	F	3.9	LE	C
3	24	79	F	2.3	B	L, C, T
3	25	74	F	3.6	LE	L, C, T
3	26	70	M	4.4	UE	L, C, T
3	27	71	M	2.2	UE	L, C, T
3	28	66	F	NA	NA	L, T
3	29	46	M	NA	NA	L, T
4	30	63	F	NA	B	L, C, T
4	31	67	F	1.1	UE	L, C, T
4	32	82	M	1.5	LE	L, C, T
4	33	75	M	1.2	B	L, C, T
4	34	60	M	1	UE	L, C, T
4	35	67	M	NA	NA	L, C
5	36	67	M	5.9	UE	L, C, T
5	37	60	F	4.6	LE	L, C, T
5	38	81	M	14.3	UE	L, C, T
NA	39	77	M	0.3	B	L, C
NA	40	39	F	1	LE	L, C, T
NA	41	NA	F	NA	NA	L, C, T

*F/M* female/male, *UE* upper extremity (cervical), *LE* Lower extremity (lumbar), *B* Bulbar, *NA* Not available, *L* lumbar, *C* cervical, *T* Thoracic

### Tissue preparation and immunohistochemistry

Spinal cord segments embedded in paraffin were sectioned at 50 µm thickness using a sliding microtome (Jung, Heidelberg, Germany), as described previously [23]. The sections were deparaffinized using xylene twice for 20 min each, which was then removed using 100% isopropanol, followed by rehydration steps beginning with 100%, 96%, 70% ethanol, and finally H_2_O. Then, the sections were incubated for 30 min in 3% hydrogen peroxide to block endogenous peroxidase activity. This was followed by an antigen retrieval step in boiling citrate buffer (pH = 6) for 20 min. Once cool, sections were blocked in 5% bovine serum albumin (BSA) and 0.25% Triton-X 100 at room temperature (RT) for 90 min and incubated on a shaking table (gentle setting) overnight at RT in primary antibodies (Table 2). The next day, the sections were rinsed 3 times in Tris-buffer and incubated for 90 min at RT with the corresponding secondary biotinylated antibody diluted in Tris-buffer. The sections were then rinsed 3 times and treated with avidin–biotin complex solution (VECTASTAIN® Elite ABC-HRP Kit, #PK-6100, Vector Laboratories, Burlingame, CA, USA) for 90 min at RT. The brown precipitate was obtained using 3,3-diaminobenzidine tetrahydrochloride (DAB) solution (D5637; Sigma, Taufkirchen, Germany). For spinal cord sections stained for pTDP-43, counterstaining for basophilic material was performed with Darrow red (Aldrich/Merck, Darmstadt, Germany) [13, 16]. Sections were dehydrated in a descending ethanol series, cleared in xylene twice, and mounted with Histomount mounting medium (National Diagnostics, Atlanta, GA, USA).

**Table 2:** List of antibodies used for immunohistochemistry (IHC)

Antibodies	Host species	Dilution	Company	Catalogue number
Synaptophysin	Mouse	1:200	Dako	M7315
phosphorylated TDP-43	Rabbit	1:5000	CosmoBio	TIP-PTD-P02
Anti-Mouse IgG Antibody (H + L), Biotinylated	Horse	1:200	Vector	BA-2000
Anti-Rabbit IgG Antibody (H + L), Biotinylated	Goat	1:200	Vector	BA-1000

The appearance of pTDP-43 pathology in different structures and regions over time was used to define the neuropathological stage based on a published staging protocol [8]: In stage 1, pTDP-43 inclusions were found in the agranular motor cortex, brainstem motor nuclei of cranial nerves V, VII, and X-XII, and in spinal cord MN. Stage 2 was characterized by involvement of the prefrontal neocortex, brainstem reticular formation, precerebellar nuclei, and the red nucleus. In stage 3, pTDP-43 pathology was present in the prefrontal cortex, postcentral neocortex, and striatum. Finally, stage 4 cases showed pTDP-43 inclusions in the anteromedial temporal lobe, including the dentate fascia and other components of the hippocampal formation. In a further step, we assessed the presence and distribution pattern of pTDP-43 inclusions in the spinal cord of all cases based on a previous staging protocol [7].

### Microscopy and image analysis

Sections stained for synaptophysin were imaged using a brightfield microscope (Keyence, BZ-X800, Osaka, Japan), with a 100 × oil objective, by taking 6 full-stack (step size of 0.5 µm) images of each spinal cord lamina of interest on two different spinal cord sections from each case. Laminae of interest were identified by their cytoarchitecture [18], laminae I and II as the most dorsal of Rexed’s laminae, lamina VII as the intermediate area extending laterally to the white matter of the lateral funiculus, and lamina IX at the base of the ventral horn. A binary image was then obtained using ImageJ (convert to mask function), by reducing the image’s noise (despeckle function) and applying a Gaussian blur filter (sigma = 1). The area covered by the synapses was then measured.

Sections stained with pTDP-43 and Darrow red were imaged with a 20 × objective by making a tile scan image of the ventral horn of the spinal cord (both left and right on two different sections for each case). MN were identified based on their location, size, and shape, and pTDP-43-positive MN were identified by means of cytoplasmic dash-like, skein-like, or dot-like inclusions [8]. The presence of any single inclusion subtype was sufficient to define a MN as “positive”. The number of MN and pTDP-43-positive MN were then counted manually.

### Statistical analysis

**Fig. 1:**
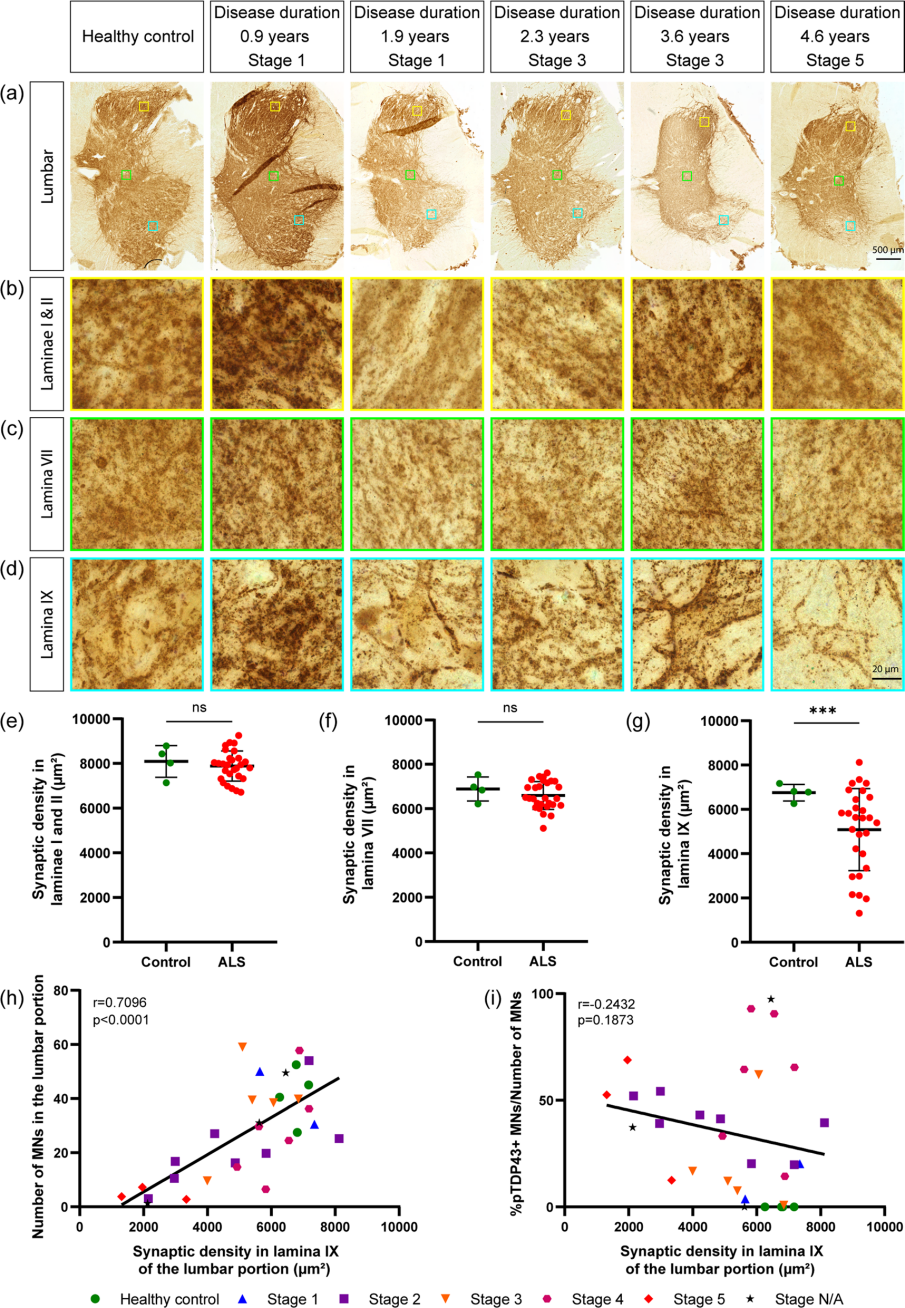
Synaptic loss in lumbar spinal cord of TDP-43-positive ALS correlates with loss of MN but not with pTDP-43-immunopositive MN. **a**–**d** Representative pictures of lumbar SC (L4-L5) overview, **a**), with high-magnification insets from laminae I-II (**b**), lamina VII (**c**) and lamina IX (**d**) from healthy control and pTDP-43 + ALS cases with different disease duration and neuropathological stage; immunostaining for Synaptophysin, 50 µm paraffin sections. Scale bar = 500 µm overview, 20 µm inset. **e–g.** Synaptic area is unchanged in laminae I-II (3) in lamina VII (**f**); in lamina IX a significant decrease in synaptic area is observed (**g**; uncorrected *t*-test *p* = 0.086; *t*-test with Welch´s correction for different SD, *p* < 0.001). **h.** The synaptic area in lamina IX is directly correlated with the number of surviving MN (r = 0.709; *p* < 0.0001). **i** No correlation between the fraction of MN displaying pTDP-43 + inclusions and the synaptic area in lamina IX. Data information: In (**e**–**g**), data are presented as means ± SD. ns, *p* > 0.05 (*t*-test and Pearson´s and Spearman´s correlation)

**Fig. 2:**
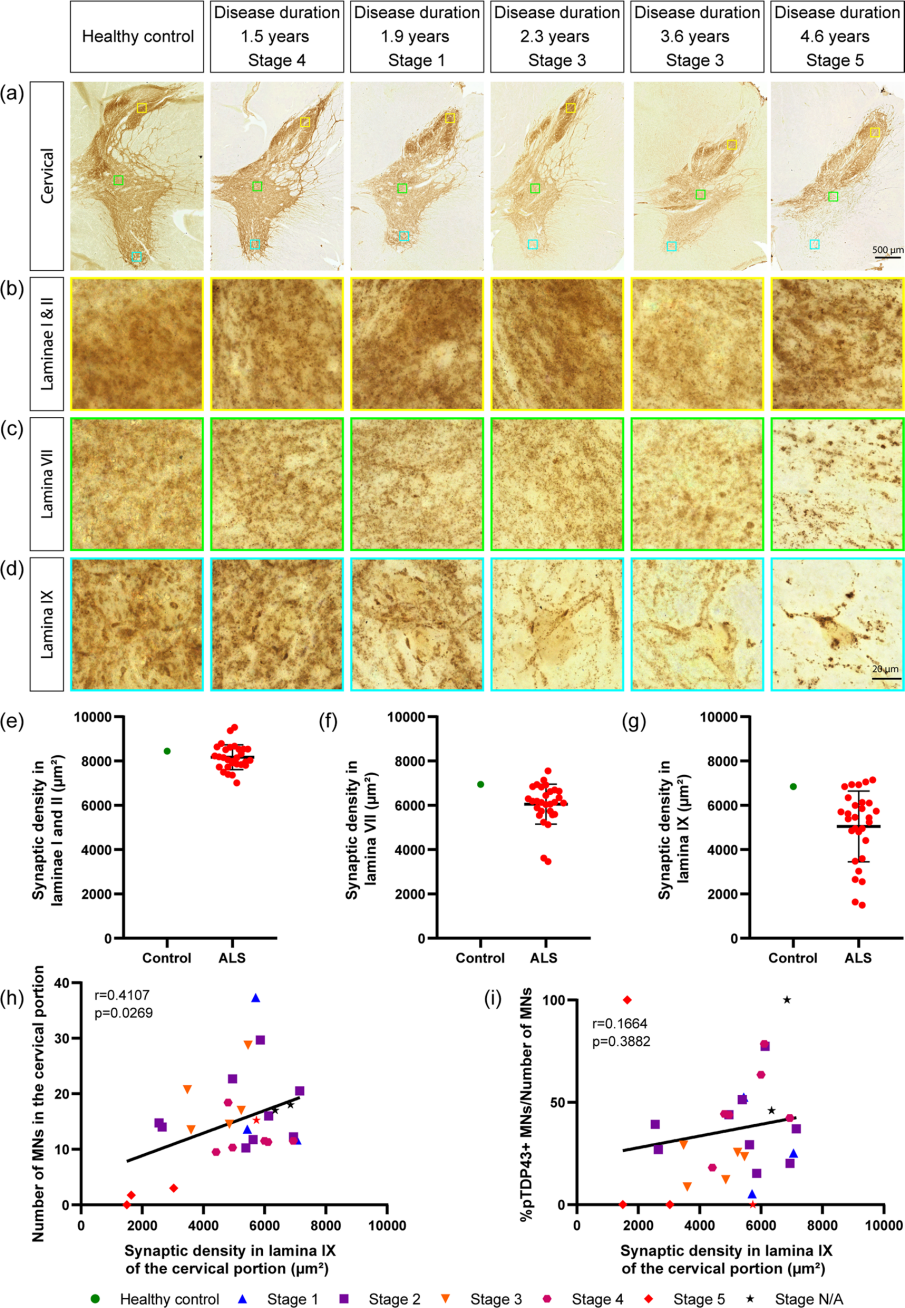
Synaptic loss in cervical spinal cord of TDP-43-positive ALS correlates with loss of MN but not with pTDP-43-positive MN. **a**–**d** Representative pictures of SC (C3-C4; overview, **a** with high-magnification insets from laminae I-II (**b**), lamina VII (**c**) and lamina IX (**d**) from healthy control and pTDP-43 + ALS cases with different disease duration and neuropathological stage; immunostaining for Synaptophysin, 50 µm paraffin sections. Scale bar = 500 µm overview, 20 µm inset. **e**–**g** synaptic area is unchanged in lamina I-II (3) and lamina VII (**f**) but shows a very strong trend toward decrease in lamina IX (**g**). **h** The synaptic area in lamina IX is directly correlated with the number of surviving MN (r = 0.410; *p* = 0.026). **i** No correlation between the fraction of MN displaying pTDP-43 + inclusions and the synaptic area in lamina IX (Pearson´s correlation). Data information: In (**e**–**g**), data are presented as means ± SD

**Fig. 3:**
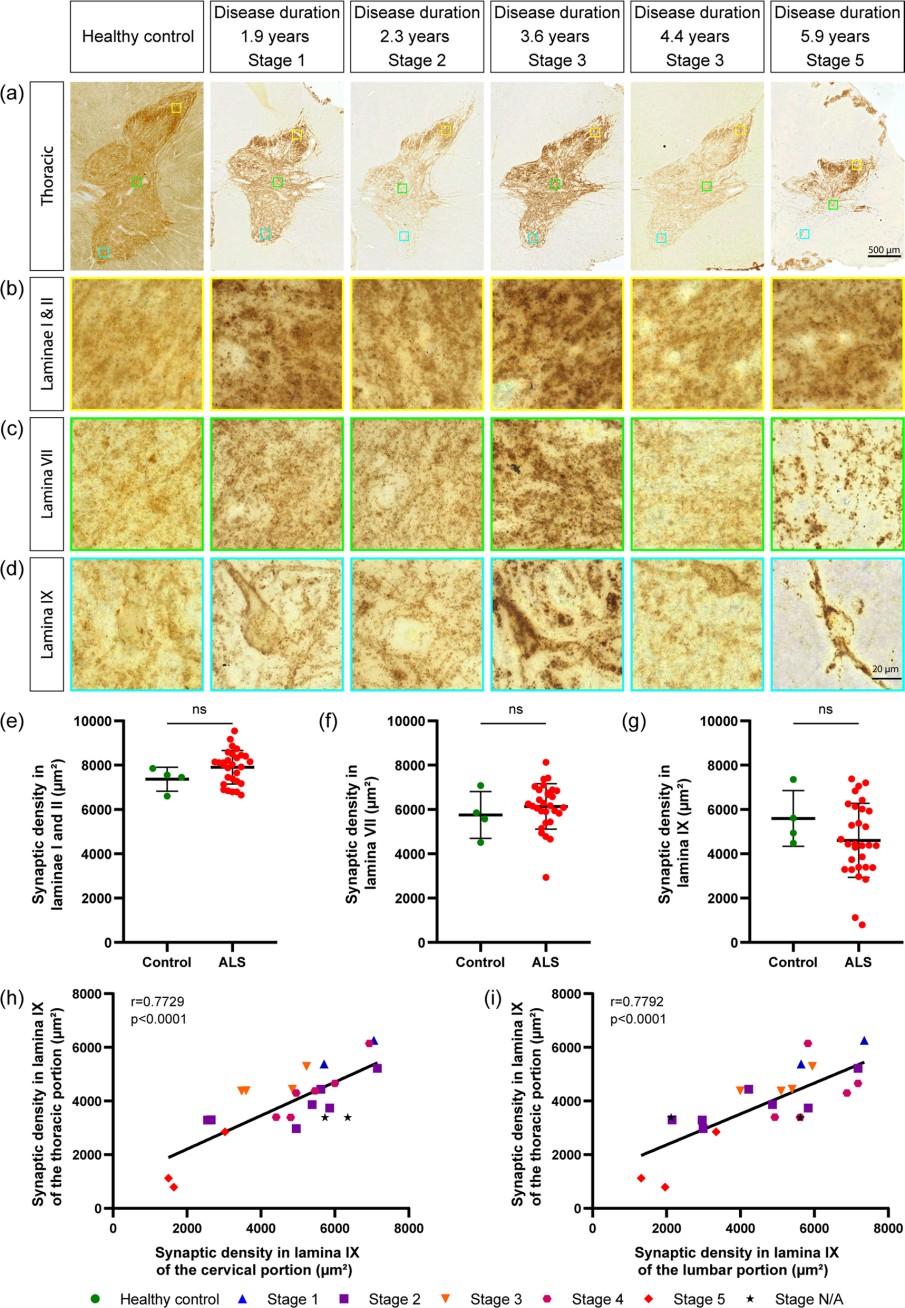
Synaptic loss in thoracic SC of TDP-43-positive ALS correlates with synaptic loss in lumbar and cervical SC. a–d Representative pictures of SC (T10-T12; overview, **a**) and with high-magnification insets from lamina I-II (**b**), lamina VII (**c**) and lamina IX (**d**) from pTDP-43 + ALS cases with different disease duration and neuropathological stage; immunostaining for synaptophysin, 50 µm paraffin sections. Scale bar = 500 µm overview, 20 µm inset. **e–g.** synaptic area is unchanged in lamina I-II (3) and lamina VII (**f**) but shows a very strong trend toward decrease in lamina IX (**g**). **h** The synaptic area in lamina IX is highly correlated with synaptic area in the lamina IX of lumbar SC. **i** The synaptic area in lamina IX is highly correlated with synaptic area in the lamina IX of cervical SC. Data information: In (**e**–**g**), data are presented as means ± SD. ns, *p* > 0.05 (*t*-test and Pearson´s correlation)

**Fig. 4:**
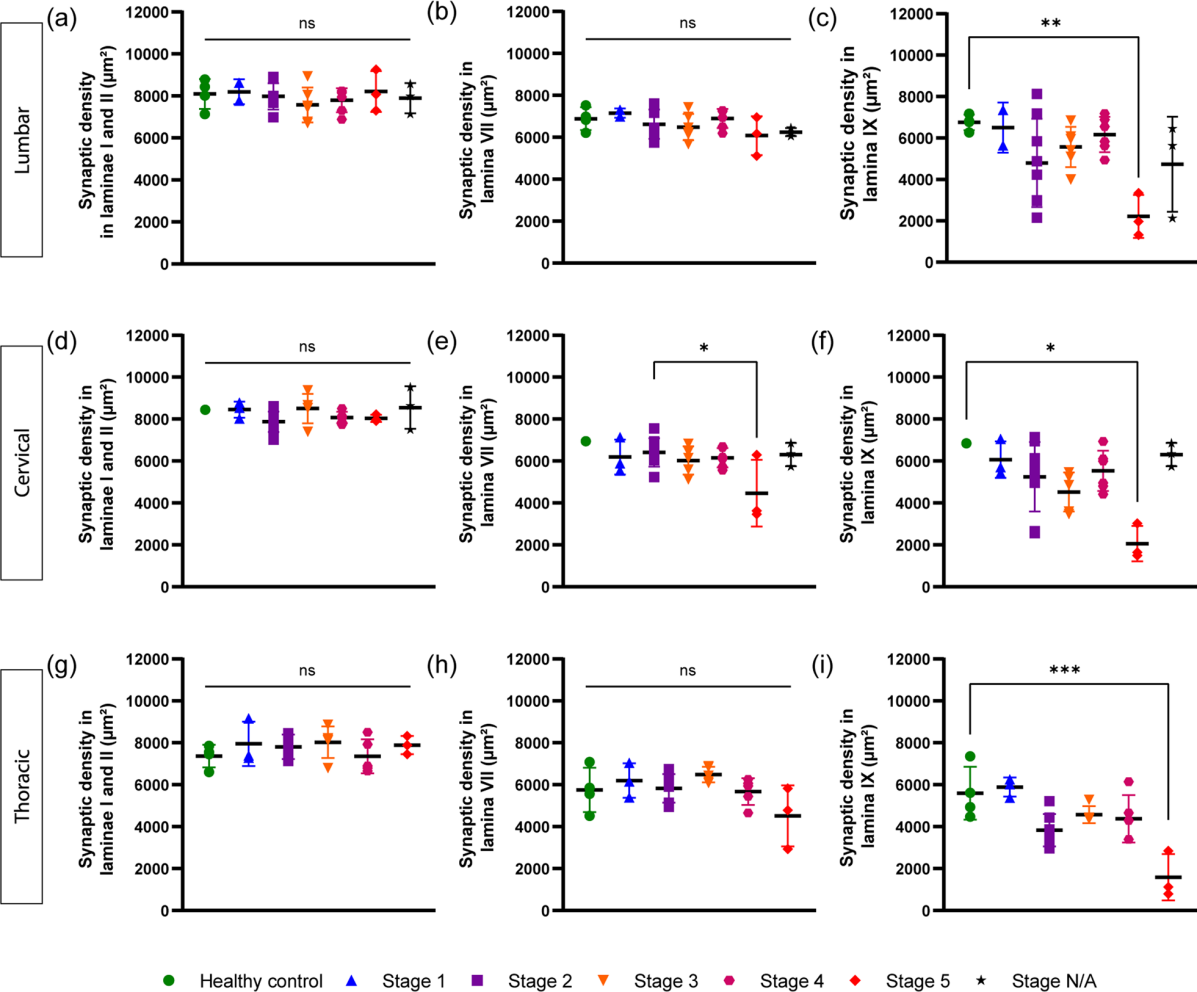
Increased Synaptic loss in cases with advanced neuropathological brain stage. **a-c.** Synaptic area in laminae I-II (**a**), lamina VII (**b**), lamina IX (**c**) of lumbar (L4-5) SC for each ALS case, subdivided according to their neuropathological brain stage. No difference is found in synaptic density in lamina II and VII across the stages; in lamina IX, a significant decrease is found for patients in stage 5, although substantial variability is observed in stages 2 to 4. **d**–**f** Synaptic area in lamina I-II (**a**), lamina VII (**b**), lamina IX (**c**) of cervical (C3-C4) SC for each ALS case, subdivided according to their neuropathological brain stage. No difference is found in synaptic density in lamina II and VII across the stages; in lamina IX, a significant decrease is found for patients in stage 5, although substantial variability is observed in stages 2–4. **g-i.** Synaptic area in laminae I-II (**g**), lamina VII (**h**), lamina IX (**i**) of thoracic (T11-12) SC for each ALS case, subdivided according to their neuropathological brain stage. No difference is found in synaptic density in lamina II and VII across the stages; in lamina IX, a significant decrease is found for patients in stage 5, although substantial variability is observed in stages 2 to 4. Each subject is depicted as a single datapoint, average of the multiple ROIs. Data information: data are presented as means ± SD. ns, P > 0.05; *, P < 0.05; **, *p* < 0.01; and ***, *p* < 0.001 (one-way ANOVA with Tukey’s test for multiple comparisons)

**Fig. 5:**
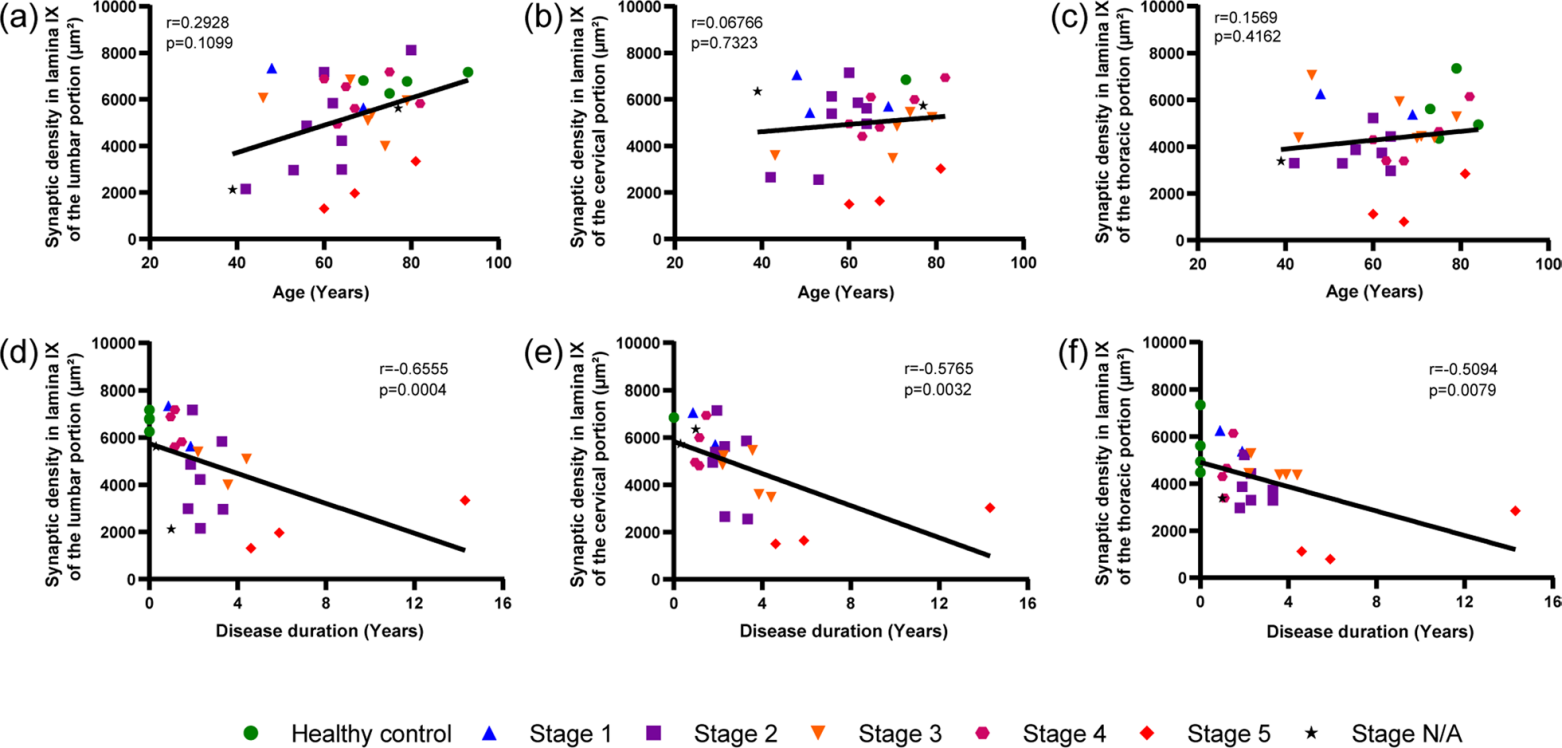
Synaptic loss in spinal cord correlates with disease duration. **a**–**c** No correlation between synaptic density and age at death in the lumbar, cervical, and thoracic spinal cord. **d**–**f** Strong correlation between synaptic loss in the spinal cord and disease duration observed in the lumbar, cervical, and thoracic spinal cord. Data information: *p* > 0.05; *, *p* < 0.05 (Pearson´s and Spearman´s correlation)

**Fig. 6:**
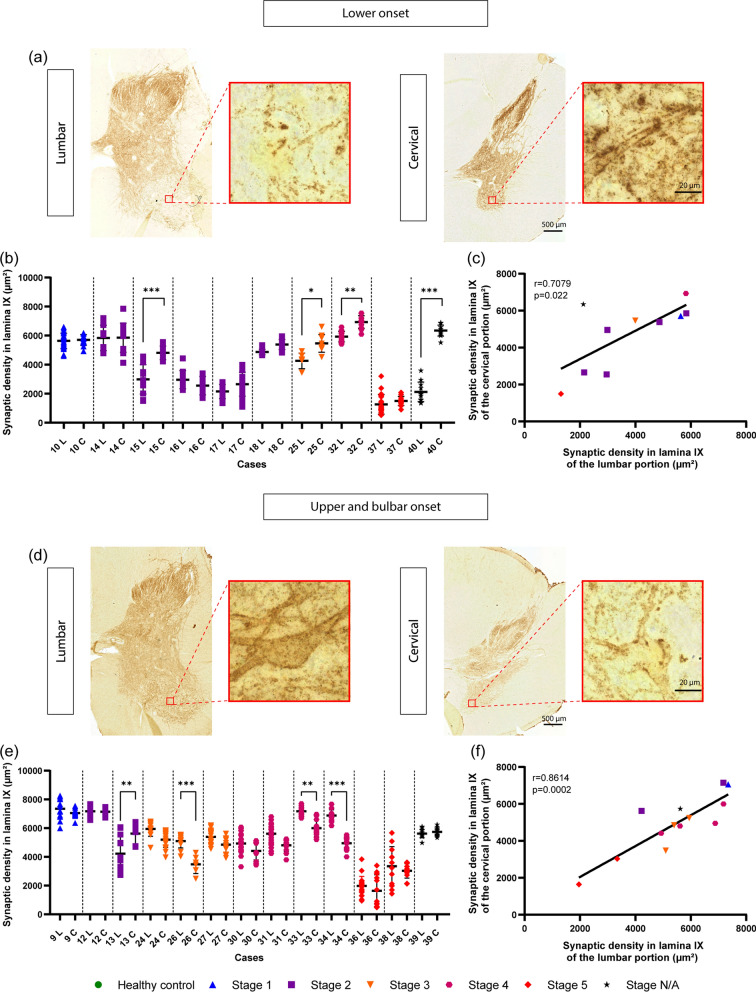
Increased loss of synapses at the site of clinical onset. **a** Representative images (synaptophysin immunoreaction in 50 µm paraffin sections) and high magnification inset of lumbar and cervical spinal cord from a case with lumbar onset (Table 1, Case No 15). **b** Synaptic area (lamina IX) in lumbar and cervical spinal cord sections from cases with lower limb onset; synaptic loss is either comparable in lumbar and cervical, or more extensive in lumbar; each datapoint represents an independent ROI in the sections, and for each subject ROIs in lumbar and cervical spinal cord are displayed. **c.** High correlation between synaptic loss in lumbar and cervical spinal cord in cases with lower limb onset. **d** Representative images and high magnification inset of lumbar and cervical spinal cord from a case with upper limbs onset (Table 1, Case No 26). **e** Synaptic area (lamina IX) in cervical and lumbar samples from subjects with cervical/bulbar onset; each datapoint represents an independent ROI in the sections, and for each subject ROIs in lumbar and cervical spinal cord are displayed. **f** High correlation between the synaptic loss in cervical and lumbar sections of spinal cord in subjects with upper (cervical/bulbar) onset. Data information: data are presented as means ± SD. ns, *p* > 0.05; *, *p* < 0.05; **, *p* < 0.01; and ***, *p* < 0.001 (one-way ANOVA with Tukey’s test for multiple comparisons)

All values are provided as mean ± standard deviation (SD). To assess differences in area covered by the synapses between controls and ALS cases overall, we used the *t*-test (Figs. 1e–g, 3e–g); when SD were significantly different between controls and ALS, Welch’s correction was applied (Fig. 1g). In this instance, we report the p value for the un-corrected and corrected *t*-test. To compare synaptic area coverage at different stages, data passed a normality test using the Shapiro-Wilks test, followed by one-way ANOVAs with post-hoc Tukey’s comparison tests (Figs. 4a–i, 6b, e). A Pearson’s correlation was used for correlation analysis when data passed Shapiro-Wilks’ normality test (Figs. 2i, h, 3i, h, 5c, e, f, 6c, f). A Spearman’s rank correlation coefficient was used for the correlation analysis when data did not pass normality test (Figs. 1i and 5a, b, d). *p < 0.05; **p < 0.01; and ***p < 0.001. Statistical tests were performed in Prism v.8.0.1 (GraphPad Software) and ImageJ v1.53f51.

## Results

### Loss of synapses in the spinal cord correlates with MN loss but not with pTPD-43 pathology

We considered a case series consisting of 33 ALS patients and 8 individuals without neurodegenerative disorders (healthy controls, Table 1). For each individual, clinicodemographic information were obtained and a neuropathological brain stage for ALS was determined, as previously reported [8]. We quantified the synaptic density (i.e., area covered by the synapses) in the lumbar spinal cord (L4-L5). For each tissue section, we considered multiple regions of interest (ROI) per region, in the dorsal (I-II), intermediate (VII) and ventral (IX) laminae (Fig. 1a–d). Compared to control subjects, ALS patients displayed a comparable synaptic density in the dorsal and intermediate areas (Fig. 1e and f), confirming the comparable quality of the specimens and the homogeneity in tissue processing. However, in the ventral laminae, ALS patients displayed significant synaptic loss (uncorrected *t*-test *p* = 0.08; after Welch´s correction for different SD, *p* < 0.0001) with a broad spread of synaptic area values (Fig. 1g).

To verify whether synaptic loss was related to the burden of pTDP-43 pathology or to the loss of MN in the ventral lumbar spinal cord, we quantified in tissue sections consecutive to those for synapse determination the absolute number of surviving MN and the fraction of MN that displayed pTDP-43 aggregates. We detected a very strong correlation between the number of surviving MN and the density of synapses in lamina IX (Fig. 1h; Additional file 1: Fig. S1). Surprisingly, there was no correlation between the fraction of pTDP-43-immunopositive MN and synaptic density (Fig. 1i; Additional file 1: Fig. S1). This result remained unchanged when three ALS cases with extensive neuronal and synaptic loss (thus, extremely reduced MN number and no surviving pTDP-43-positive MN) were excluded.

We performed a similar exploration in the cervical spinal cord (Fig. 2a–d). ALS patients displayed a comparable synaptic area in dorsal and intermediate laminae (Fig. 2e–f) of the C2-C3 cervical spinal cord but a significant decrease in the ventral horn (Fig. 2g), again with a wide distribution of values for individual cases. In addition, in the cervical spinal cord the synaptic area coverage correlated with the number of surviving MN (Fig. 2h; Additional file 2: Fig. S2) but not with the fraction of pTDP-43-positive MN (Fig. 2i; Additional file 2: Fig. S2).

No effect of sex on synaptic loss was observed, with male and female ALS patients displaying comparable values of synaptic loss in ventral horn of lumbar (Additional file 3a: Fig. S3) and cervical sections (Additional file 3b: Fig. S3).

We also investigated the degree of synaptic involvement of the thoracic spinal cord (T11-T12; Fig. 3a–d)). The overall synaptic area in dorsal, intermediate, and ventral laminae was comparable in ALS and control cases (Fig. 3e–g). Notably, synaptic area in lamina IX of thoracic spinal cord was highly correlated with values of synaptic area in lamina IX of the corresponding lumbar (Fig. 3h) or cervical sections (Fig. 3i), indicating that, although not statistically significant in the direct contrast *versus* control cases (Fig. 3g), synaptic loss in thoracic lamina IX follows the same trend observed in the cervical and lumbar spinal cord samples from the same patients.

Next, we looked to see if the loss of synapses was related to the overall progression of pTDP-43 pathology in the brain, as revealed by the neuropathological brain stage (available for 30 out of 33 samples, with three additional samples classified as “inconclusive”). In the dorsal and intermediate laminae, no difference was found across neuropathological stages (Fig. 4a, b). In the ventral horn (Fig. 4c), ALS stage 1 cases (n = 2) were comparable to controls, but patients with ALS stage 2, 3, and 4 displayed synaptic area ranging from normal values to substantially decreased. Only the subjects with ALS stage 5 pathology (n = 3) [7] displayed a statistically significant loss of synaptic area compared to controls (Fig. 4c). Likewise, in the cervical spinal cord, no difference was observed in the dorsal and intermediate laminae (Fig. 4d, e) across all ALS neuropathological stages; synaptic area in ventral horn was significantly reduced only in stage 5 cases (Fig. 4f). Same observation in the thoracic spinal cord, where no difference was found across the subgroups, except for the stage 5 samples that differed significantly from healthy subjects (Fig. 4i).

Thus, the loss of synapses in the spinal cord of ALS patients was restricted to the ventral horn and correlated with the loss of MN but not with the burden of pTDP-43 pathology. Moreover, as predicted by the propagation model of pTDP-43 spreading [6, 14], the extent of synaptic loss in ALS patients was stage-independent [8].

### Disease duration predicts synaptic loss in lamina IX

We then examined the chronological pattern of synaptic loss in the ALS spinal cord. We correlated the synaptic density in cervical, thoracic, and lumbar spinal cord samples with the age of the patients at death and with disease duration. Synaptic density did not correlate with age at death, indicating a minimal effect of aging itself on synaptic loss (Fig. 5a–c). On the other hand, a strong inverse correlation between synaptic density and disease duration was found in lumbar, thoracic, and cervical samples (Fig. 5d–f). Inclusion or exclusion of healthy individuals, as well as inclusion or exclusion of a single patient with a long disease duration (case No 38), did not alter these findings.

### Site of clinical symptoms onset is associated with more severe synaptic loss in lamina IX

We also considered whether a more pronounced synaptic loss characterized the site of clinical symptom onset.

First, we investigated whether the presence of upper MN symptoms/signs resulted in a more pronounced synaptic loss. We took into consideration a subset of n = 24 patients for whom symptoms/signs related to lower MN or upper MN were recorded in clinical charts. We divided the patients into two subgroups, i.e., those displaying predominantly lower MN symptoms/signs and those displaying both upper and lower MN symptoms/signs (no patient in our cohort displayed only upper MN symptoms/signs). The extent of synaptic loss in the ventral horn of the lumbar spinal cord as well as cervical spinal cord was comparable in the two subgroups (Additional file 4: Fig. S4, thereby suggesting that the clinically-detectable upper MN involvement did not alter the overall extent of synaptic loss in spinal cord ventral horn.

We then considered a subset of n = 23 patients for whom detailed clinical data were available and divided them into “lumbar onset” and “bulbar or cervical onset” (no thoracic onset patient was available) and compared the synaptic density (lamina IX) in the cervical *versus* lumbar spinal cord for each group (Fig. 6). Among the 10 patients with lumbar onset (Fig. 6a and b), four displayed a significantly lower synaptic density in the lumbar *versus* cervical cord. The remaining six showed a comparable loss in the cervical and lumbar cord. For at least three cases, synaptic density values were severely reduced in both cervical and lumbar sections (hindering the comparison because of possible floor effects). In no case with lower onset the synaptic density was more compromised in cervical than in lumbar spinal cord.

Conversely, among the 13 patients with bulbar/cervical onset (Fig. 6d and e), most of them displayed larger burden in cervical than in lumbar, 4 displayed a strong trend in this direction, 3 showed no difference and 2 had a profound synaptic loss both in cervical and lumbar (possible floor effect). Remarkably, only one patient (case 13) displayed a more severe synaptic loss in lumbar than in cervical. Nevertheless, both in patients with upper onset and in those with lower onset, synaptic density in the ventral horn in cervical and lumbar samples was highly correlated, respectively (Fig. 6c and f). Taken together, these findings are consistent with disease propagation within local synaptic networks in the spinal cord as function of disease duration.

## Discussion

In the present study involving 41 spinal cord specimens from n = 8 healthy subjects and n = 33 ALS patients, we found that synapses were extensively lost in the ventral horn of the spinal cord of ALS patients but not in controls. Synaptic loss correlated with the disease duration and the loss of MN but not with the local fraction of pTDP-43-positive MN. The extent of synaptic loss and the fraction of pTDP-43 were not related to the extent of cortical spread of pTDP-43 pathology.

Our finding related to the loss of synapses in the ventral spinal cord is largely in agreement with previous studies based on substantially smaller cohorts. Reduced density of synapses on MN dendrites, although associated with increased synaptic size, was reported by Sasaki et al. 1995 using ultrastructural methods on spinal cord samples from four ALS patients and seven controls and, again, in a cohort of five ALS patients and five controls [33] in agreement with earlier reports [26]. Likewise, loss of synaptophysin and synapsin immunoreactivity (but not of SNAP25 or syntaxin) was reported by Ikemoto et al. [20], who compared five ALS cases and eight control individuals. Moreover, in a comparison of 11 ALS patients and 40 controls, a distinct loss of synapses was detected [12]. A small, but consistent decline in overall synaptic density was detected in the ventral spinal cord of ALS patients as well as in patients affected by progressive muscular atrophy [21]. A decline in the number of synapses has been also reported in the contrast of the cervical ventral horn of six control subjects and nine ALS cases; in that study, a significant synaptic loss was seen in the C9Orf72 subgroup but not in the SOD1 subgroup [9]. Thus, synaptic loss in the ventral horn of the ALS spinal cord has been consistently demonstrated, although the small cohort sizes of these studies prevented the full appreciation of the range of synaptic loss in ALS. A major strength of the present study lies in the large cohort with characterized disease courses, which enabled clinicopathological correlations.

Of note, reduced synaptic density and altered presynaptic composition have also been observed in iPSC-derived MN with multiple mutations and in synaptic proteomes from pathological specimens [11, 35]. Likewise, synaptic alterations have been reported in murine models of ALS both in the presymptomatic [4] and early symptomatic [9] phases. Our findings thereby confirm that in vitro models and animal models may recapitulate in vivo human neuropathology.

Notably, an association between the structural abnormalities or loss of synapses and initial signs of MN degeneration (defined as chromatolysis, the loss of Nissl substance; [12, 25], has been reported [12, 32], thereby indicating that MN dysfunction may be causally related to synaptic loss on individual MN [32]. Our findings are compatible with this interpretation inasmuch as the large-scale synaptic loss correlated with MN loss – although, given the substantial loss of synapses observed in some cases, we cannot exclude a contribution originating from the dysfunction or loss of other ventral horn neurons. Of note, expansion of the size of remaining synaptic contact has been previously reported [11, 32]; the “synaptic area” readout used in the present study integrates both number and size of synapses (since the immunohistochemical technique employed does not always allow for the resolution of individual synaptic boutons) and may therefore be less sensitive in the early phases of the disease, when loss of synapses may be compensated by the expansion of the size of the remaining ones.

Interestingly, the fraction of TDP-43-positive MN did not correlate with the extent of large-scale synaptic loss. One possible interpretation of this finding is that synaptic loss increases the vulnerability of pTDP-43-positive MN to cell death, accelerating their elimination and therefore preventing their appearance in the histological quantification. This model is in agreement with the role of synaptic activity in delivering neuroprotective signals in several types of neurons [4, 5, 11, 31]. When the role of pTDP-43 itself is in regulating synaptic function [28] as well as in the splicing of synaptic genes [10], it is possible that a positive feedback loop between synaptic loss and pTDP-43 pathology may account for the runaway loss of pTDP-43-positive MN synaptic loss upon synaptic dysfunction in ALS. Moreover, non-cell-autonomous mechanisms, possibly involving microglial reactivity, may also influence the large-scale loss of synapses [30].

Overall, the progression of synaptic loss in the ALS spinal appears not related to the propagation of pTDP-43 pathology to cortical areas; this is compatible with the overall architecture of corticospinal projections since most cortical areas affected by the pTDP-43 pathology do not have strong projections to the spinal cord. In this respect, an important unanswered question that requires additional studies is whether, at some point during the disease course, synaptic density loss in the spinal cord of ALS patients proceeds, in some way independently of the initial disease process in the agranular motor cortex. Our findings relative to stage 1 patients (whose samples were only available in small numbers) show little if any large-scale synaptic loss. However, it is possible that a small degree of synaptic loss or an alteration affecting primarily the post-synaptic structures of MN may not have been detected in the present study.

The present study is not without limitations. First, the autopsy samples for this cross-sectional study were collected over a time span of several decades and therefore were subject to different storage conditions for fixed tissue, which in turn may have reduced synaptophysin immunostaining efficacy. To counteract this potential limitation, we used both the dorsal laminae and the thoracic spinal cord samples as internal benchmarks to avoid the inclusion of damaged or suboptimally archived samples. The same applies to the availability and quality of brain tissue samples for neuropathological staging: three of the ALS cases studied could not be staged with certainty, because not all of the required tissue blocks were available (Table 1, cases 39–41). In addition, for five ALS patients, the age at death, gender, and site of clinical onset were unavailable (Table 1, cases 11, 19–22, 35). For control cases, cervical spinal cord samples of suitable quality were available for only one case; extensive sampling of the spinal cord is not performed routinely unless there is clinical indication, which did not exist for control cases (per definition). Second, determination of the site of disease onset was based on clinical reports and patients’ complaints. As a result, the site of onset may have been misreported by some patients if their initial symptoms were overlooked, thereby leading to an incongruence between the site of onset and the extent of synaptic loss. A clinicopathological study in a prospective cohort would be needed to address this point.

In conclusion, we could show that synaptic loss in the spinal cord ventral horn of ALS patients is highly variable with regard to the degree of its severity, which in turn relates to the site of clinical onset within the spinal cord but not to the presence of upper MN involvement (see also [7]) and correlates with the duration of disease, reflecting a pathogenic process over time.

## Supplementary Information

**Additional file 1: Fig. S1**. pTDP-43 pathology in the lumbar portion of the spinal cord Representative images of the ventral horn of the spinal cord with high-resolution images of pTDP43-negative MN for the control and pTDP43-positive MNs for ALS cases from different stages in 50 µm paraffin sections. Scale bar = 200 µm; inset 20 µm.

**Additional file 2: Fig. S2**. pTDP-43 pathology in the cervical portion of the spinal cord. Representative images of the ventral horn of the spinal cord with high-resolution images of pTDP43-negative MN for pTDP43-positive MNs for ALS cases from different stages in 50 µm paraffin sections. Scale bar = 200 µm; inset 20 µm.

**Additional file 3: Fig. S3**. Comparable synaptic loss in male and female ALS patients. **a** Synaptic area in the ventral horn of lumbar spinal cord was comparable in male and female ALS patients. **b** Synaptic area in the ventral horn of cervical spinal cord was comparable in male and female ALS patients. ns = *p* < 0.05 (*t*-test). Data information: In (**a** and **b**), data are presented as means ± SD.

**Additional file 4: Fig. S4**. Similar synaptic loss in patients with predominant lower MN symptoms/signs or with both upper (cortical) and lower (spinal) MN involvement. The overall synaptic area in the ventral horn of lumbar and cervical spinal cord was comparable in ALS patients displaying predominantly lower MN (LMN) signs and in patients displaying both upper MN (UMN) signs and LMN signs. ns = *p* < 0.05 (*t*-test). Data information: data are presented as means ± SD.

## Data Availability

The datasets used and/or analyzed during the current study are available from the corresponding author by reasonable request.

## Abbreviations

ALS: Amyotrophic lateral sclerosis
MN: Motoneuron
pTDP-43: Phosphorylated TAR DNA-binding protein
CST: Corticospinal tract
DAB: Diaminobenzidine tetrahydrochloride
SOD: Superoxide dismutase
iPSC: Induced pluripotent stem cells
SNAP25: Synaptosome associated protein 25
C9Orf72: Chromosome 9 open reading frame 72
