# Supplementary figures and images for: Patterns of synaptic loss in human amyotrophic lateral sclerosis spinal cord: a clinicopathological study
Source: Acta Neuropathol Commun. 2023 Jul 25;11:120. doi: 10.1186/s40478-023-01616-8 (PMC10367350; doi:10.1186/s40478-023-01616-8)

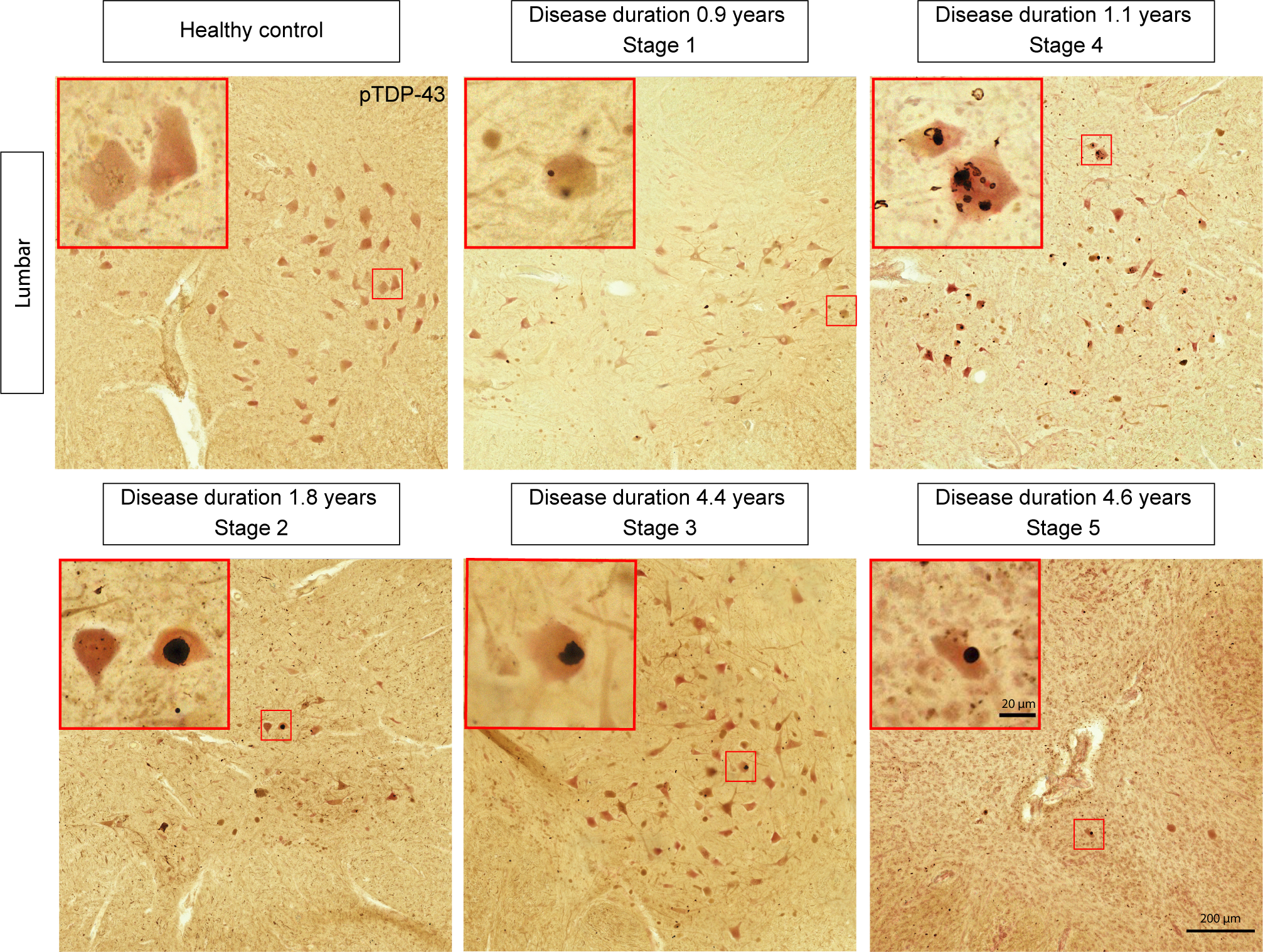

Supplement: Supplementary file 1 — Additional file 1: Fig. S1. pTDP-43 pathology in the lumbar portion of the spinal cord Representative images of the ventral horn of the spinal cord with high-resolution images of pTDP43-negative MN for the control and pTDP43-positive MNs for ALS cases from different stages in 50 µm paraffin sections. Scale bar = 200 µm; inset 20 µm. [file 40478_2023_1616_MOESM1_ESM.tif]

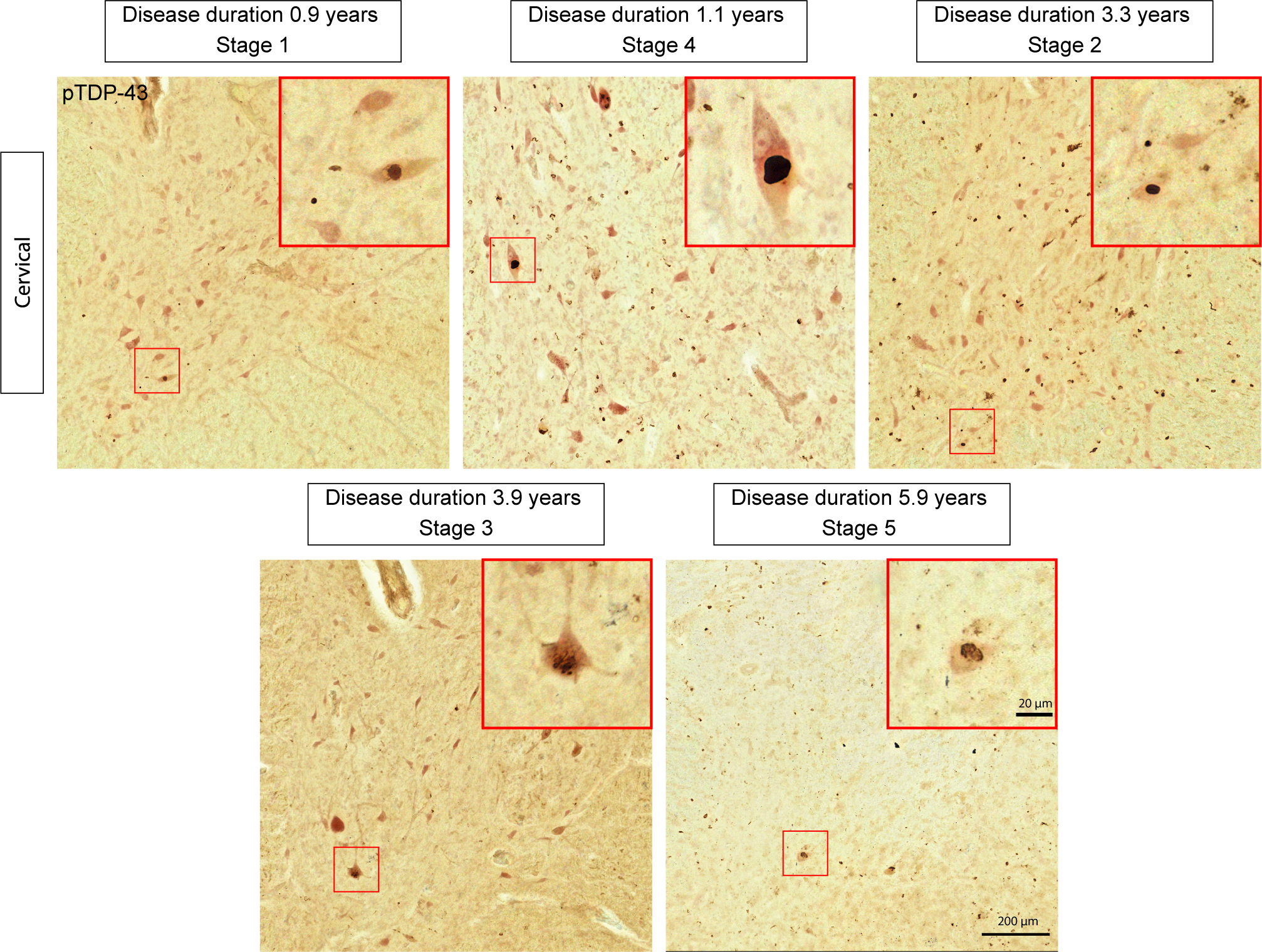

Supplement: Supplementary file 2 — Additional file 2: Fig. S2. pTDP-43 pathology in the cervical portion of the spinal cord. Representative images of the ventral horn of the spinal cord with high-resolution images of pTDP43-negative MN for pTDP43-positive MNs for ALS cases from different stages in 50 µm paraffin sections. Scale bar = 200 µm; inset 20 µm. [file 40478_2023_1616_MOESM2_ESM.tif]

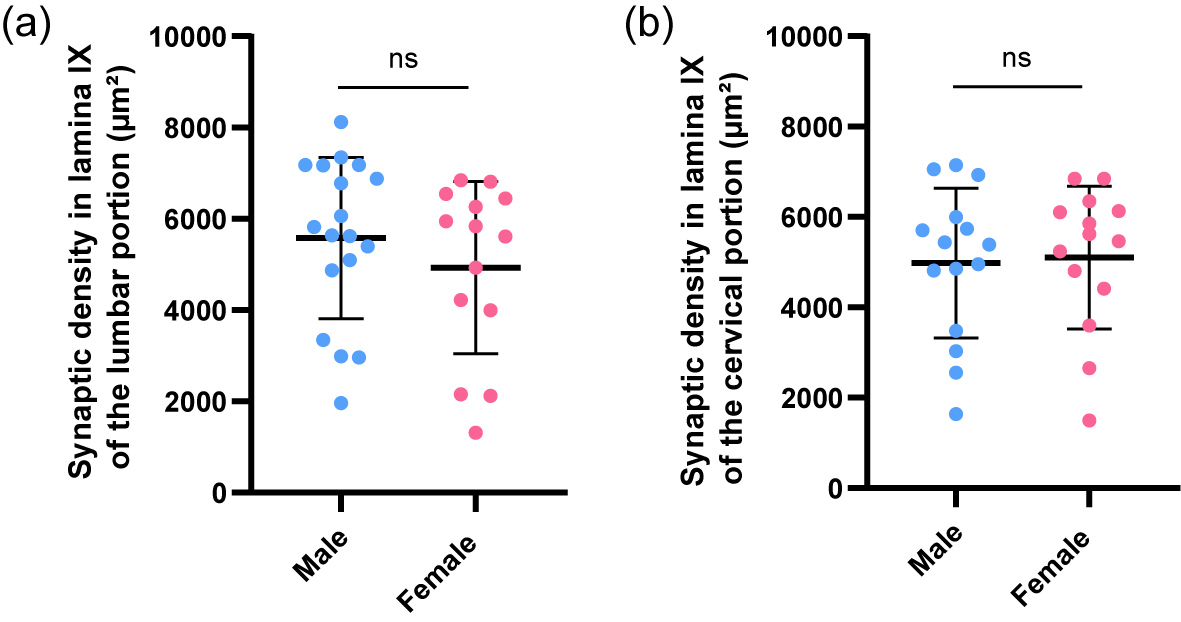

Supplement: Supplementary file 3 — Additional file 3: Fig. S3. Comparable synaptic loss in male and female ALS patients. a Synaptic area in the ventral horn of lumbar spinal cord was comparable in male and female ALS patients. b Synaptic area in the ventral horn of cervical spinal cord was comparable in male and female ALS patients. ns = p < 0.05 (t-test). Data information: In (a and b), data are presented as means ± SD. [file 40478_2023_1616_MOESM3_ESM.tif]

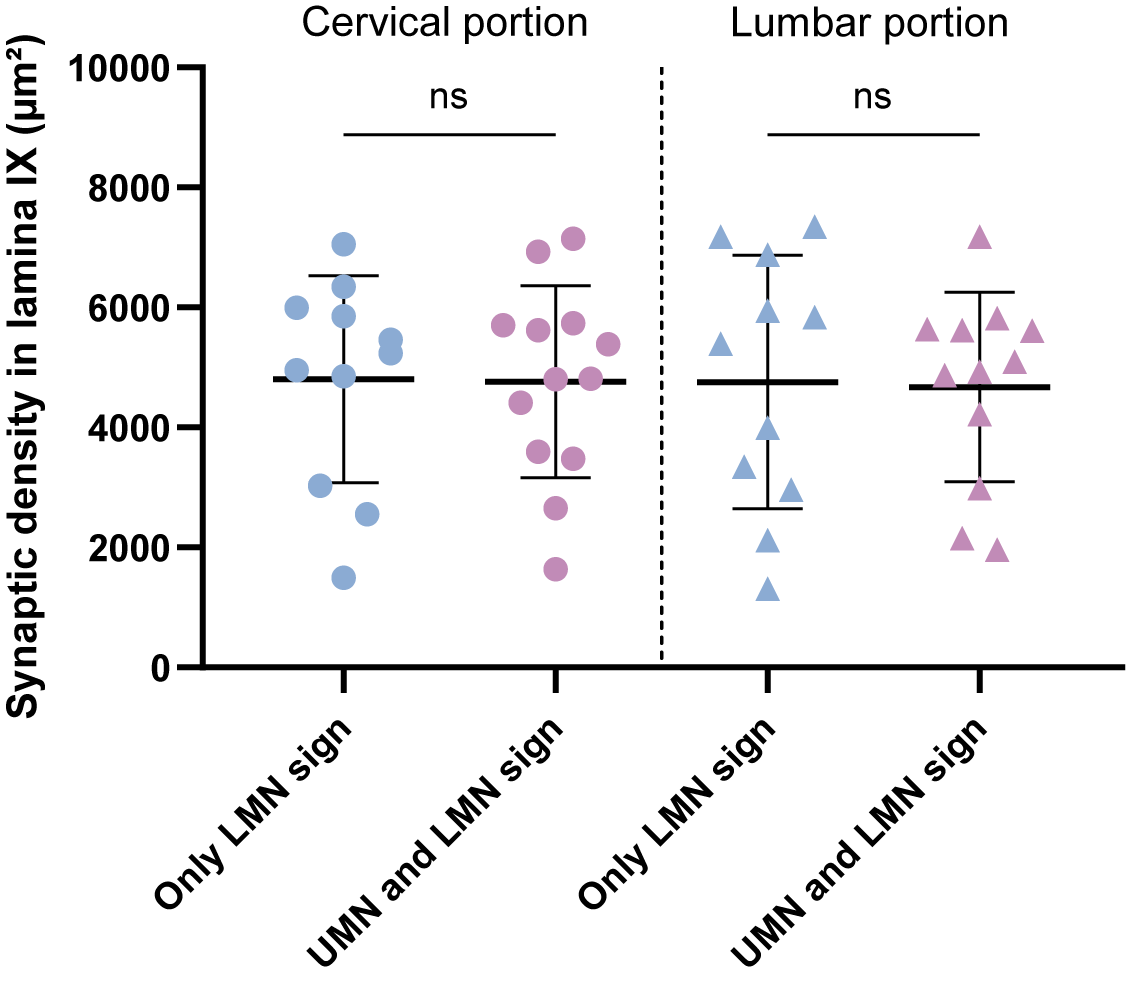

Supplement: Supplementary file 4 — Additional file 4: Fig. S4. Similar synaptic loss in patients with predominant lower MN symptoms/signs or with both upper (cortical) and lower (spinal) MN involvement. The overall synaptic area in the ventral horn of lumbar and cervical spinal cord was comparable in ALS patients displaying predominantly lower MN (LMN) signs and in patients displaying both upper MN (UMN) signs and LMN signs. ns = p < 0.05 (t-test). Data information: data are presented as means ± SD. [file 40478_2023_1616_MOESM4_ESM.tif]
